# Differences and Correlations of Anxiety, Sleep Quality, and Pressure-Pain Threshold between Patients with Chronic Low Back Pain and Asymptomatic People

**DOI:** 10.1155/2022/8648584

**Published:** 2022-05-17

**Authors:** Changming Xu, Zhiwei Fu, Juan Wang, Bao Wu, Xue-Qiang Wang

**Affiliations:** ^1^Department of Rehabilitation, Renji Hospital Affiliated to Shanghai Jiaotong University School of Medicine, Shanghai, China; ^2^Department of Sports Rehabilitation, Shanghai University of Sport, Shanghai, China; ^3^Department of Bone and Joint Surgery, Renji Hospital Affiliated to Shanghai Jiaotong University School of Medicine, Shanghai, China; ^4^Department of Rehabilitation Medicine, Changzhou Geriatric Hospital Affiliated to Soochow University, Changzhou, China; ^5^School of Health and Rehabilitation, Jiangsu College of Nursing, Huaian, China; ^6^Department of Rehabilitation Medicine, Shanghai Shangti Orthopedic Hospital, Shanghai, China

## Abstract

**Background:**

Chronic low back pain (CLBP) is a clinically common and expensive disease. Patients frequently take sick leaves because of pain and dysfunction, and their unpleasant life and work experiences cause psychological depression and anxiety and affect their quality of life. Sleep disturbance is a common problem among patients with low back pain (LBP) with more than 50% complaining about poor sleep quality. This study aimed to explore the correlations between anxiety, sleep quality, and pressure-pain threshold (PPT) and their differences between patients with CLBP and asymptomatic people.

**Methods:**

Forty patients with CLBP and 40 asymptomatic people were recruited. Relevant data, including State-Trait Anxiety Inventory, Pittsburgh Sleep Quality Index, and PPT, were individually and independently collected by blinded physiotherapists with a practicing certificate and then statistically analyzed. An independent sample *t*-test was used to determine the intergroup differences between patients with CLBP and asymptomatic populations. Pearson correlation coefficient was employed for correlation analysis.

**Results:**

The CLBP group had significantly higher anxiety scores (41.64 ± 9.88 vs. 36.69 ± 8.31; *t* = −2.496, *p*=0.015) than the asymptomatic group. A significant difference was found in the total score of the Pittsburgh Sleep Quality Index (6.41 ± 2.43 vs. 5.09 ± 2.18; *t* = −2.628, *p*=0.010) but not in the trait anxiety (44.00 ± 7.83 vs. 42.67 ± 9.51; *t* = −0.695, *p*=0.489) of the two groups. State−Trait Anxiety Inventory showed a low to moderate negative correlation with PPT. No remarkable correlation was observed between Pittsburgh Sleep Quality Index and PPT.

**Conclusions:**

Patients with CLBP showed considerably worse state anxiety and sleep quality than asymptomatic people; however, no substantial difference in PPT was found between the two groups. The results suggest that in clinical practice, the focus should include pain and related social and psychological factors. CLBP treatment could be considered from multiple perspectives and disciplines.This trial is registered with Chinese Clinical Trial Registry (Trial registration: ChiCTR-TRC-13003701).

## 1. Introduction

Chronic low back pain (CLBP) is a clinically common and expensive disease [1, 2]. Its long course has generated huge medical expenditures in various countries [3]. When low back pain (LBP) lasts for more than 3 months, it is no longer regarded as a symptom but rather a disease caused by the interaction of factors that may differ from the initial cause [3]. LBP is usually caused by injury [4]. Spinal structural instability and intervertebral disc herniation can also occur in asymptomatic individuals. Therefore, degenerative changes in spinal structure and stability are not directly related to pain pathology [5–7]. A biopsychosocial model that includes related biological and nonbiological risk factors, such as negative beliefs about pain, anxiety, depression, emotional confusion, painful behaviors, and sleep quality, is suitable for clinical practice [8–10].

LBP usually does not manifest alone and may be accompanied by pain in other body parts [11, 12]. Patients frequently take sick leaves because of pain and dysfunction [13], and their unpleasant life and work experiences cause psychological depression and anxiety and affect their quality of life [14]. Chronic pain and anxiety coexist and may cause each other, forming a vicious cycle [15]. Some scholars [15, 16] designed a patient-centered treatment model that focuses on the biopsychosocial factors of patients. They found improvements in pain intensity, disability, quality of life, and self-efficacy of patients after the intervention compared with the control, but anxiety and stress were less ameliorated. On this basis, the correlation between psychosocial factors and CLBP must be further investigated.

Sleep disturbance is a common problem among patients with LBP with more than 50% complaining about poor sleep quality [17–19]. Reduced sleep disrupts the complete sleep cycle and may increase musculoskeletal pain and sensitivity to harmful stimuli [20]. Pain intensity and anxiety score can be enhanced by improving sleep quality in patients with chronic pain [21, 22]. Anxiety may also be related to pain intensity and sleep quality in patients with fibromyalgia [23]. Patients with CLBP may simultaneously experience anxiety, poor sleep quality, and pain, and these three affect each other.

Muscle pressure-pain threshold (PPT) refers to the pressure threshold at which a subject experiences pain [24]. Peripheral or central sensitization occurs with changes in pain intensity [25]. Moderate pain may cause changes in PPT and adaptations of the musculoskeletal system [26, 27]. Therefore, it can effectively provide objective values about changes in pain sensitivity in patients with CLBP [28]. Moreover, PPT is often used as an evaluation tool to evaluate the effect of related interventions on LBP in the research on the pain sensitivity of exercise [29–33]. However, few studies investigated whether patients with CLBP and asymptomatic people have differences in PPT [24–30]. Whether the changes in the muscle PPT of patients with CLBP are the result of anxiety or sleep disturbance or a factor that causes pain also remains unclear. This study aimed to explore the differences and correlations among anxiety, sleep quality, and PPT and their differences between patients with CLBP and asymptomatic people.

## 2. Materials and Methods

The relevant procedures and plans were reviewed and approved by the Ethics Committee of Shanghai University of Sports and then registered at the Chinese Clinical Trial Registry Trial registration: ChiCTR-TRC-13003701. The experiment was carried out at the Sports Rehabilitation Center of Shanghai University of Sports and the Rehabilitation Department of Renji Hospital affiliated with Shanghai Jiaotong University School of Medicine. Participants were recruited through online and offline methods, and the experiment was initiated after the patients signed the informed consent form.

### 2.1. Subjects

Among the 92 subjects recruited, 12 did not meet the inclusion criteria. Finally, 40 subjects with CLBP and 40 asymptomatic people were included. The sample size was calculated as previously described [26].

The inclusion criteria for the CLBP group were as follows: (1) 18–50 years old, (2) LBP for at least 3 months, (3) numerical rating scale (NRS) score ≥3, (4) Roland Morris Disability Questionnaire score ≥3, and (5) those that can sign the informed consent and complete relevant tests.

The inclusion criteria for the asymptomatic group were as follows: (1) 18–50 years old; (2) no history of LBP and no pain in other body parts during recruitment; and (3) those that can sign the informed consent and complete relevant tests.

The exclusion criteria for both groups were as follows: (1) currently taking analgesics and anesthetics; (2) addicted to smoking or alcohol; (3) cognitive impairment or psychological illness; (4) history of surgery; (5) current treatment for other clinical diseases, such as fractures, sprain, and cold; and (6) pregnant women.

Patients were withdrawn if (1) the subjects personally request a withdrawal from the study, (2) the subject cannot complete the test as required, (3) the symptoms of LBP worsened, or (4) the subject received other treatment.

### 2.2. Procedure

After recruitment, an equal number of subjects were recruited into the CLBP and asymptomatic groups. Relevant data, including State-Trait Anxiety Inventory, Pittsburgh Sleep Quality Index, and PPT, were individually and independently collected by blinded physiotherapists with a practicing certificate and then statistically analyzed.

### 2.3. Outcome Measures

Baseline data consist of demographic information (age, gender, height, weight, and education) and self-reported physical activity (sitting for a long time; exercising regularly; and the duration, weekly frequency, and intensity of exercise). State-Trait Anxiety Inventory and Pittsburgh Sleep Quality Index were then measured.

PPT data (kgf) were collected using a handheld PPT tester (FDX 25 FORCE GAGE 25 × 0.02 Ibf) to apply pressure at a constant speed on specific muscle positions. The pressure stimulation was immediately stopped when the subject reported pain, and the corresponding value was recorded. The measurement was repeated four times for each position and then averaged. Prior to formal data collection, the subjects were familiarized with the measurement procedure in advance. Measurement was generally performed on the painful side of the subjects. If the pain site was symmetrical, then the dominant side was measured.

The test points in the waist [26] included the iliopsoas, quadratus lumborum, erector spinae, transversus abdominis, gluteus medius, and piriformis muscles. The proximal measurement positions included the levator scapula and rhomboid muscles. The distal measurement positions included the hamstring and gastrocnemius muscles.

### 2.4. Statistical Analysis

Microsoft Excel 2019 (Microsoft, Redmond, WA, USA) and IBM SPSS 20.0 (IBM Corp., Armonk, NY, USA) were used for data statistics and analysis. An independent sample *t*-test was applied to determine the intergroup differences between patients with CLBP and asymptomatic populations. The results were expressed as mean (*M*) ± standard deviation (SD) with corresponding *t* and *p* values.

Pearson correlation coefficient was employed for correlation analysis, and the statistical results indicate the Pearson coefficient and corresponding *p* value. *p* < 0.05 was considered significantly different. An absolute value of the Pearson coefficient close to 1 indicates a strong correlation: 0.00–0.19 indicates very low correlation, 0.20–0.39 indicates low correlation, 0.40–0.69 indicates moderate correlation, 0.70–0.89 indicates high correlation, and 0.90–1.00 indicates extremely high correlation.

## 3. Results

Among the 92 recruited people, 12 did not meet the inclusion criteria and were excluded at the initial screening. Eighty subjects participated in the data collection. Subject matching at a ratio of 1 : 1 was adopted during grouping to exclude the influence of gender, age, and other factors. The patients with CLBP and asymptomatic populations showed consistent gender composition with no differences in baseline data.

### 3.1. Baseline Data

Baseline data included age, gender, height, weight, education, and self-reported exercise habits (including sedentary or not, regular training or not, duration of each training, weekly training frequency, and training intensity). No remarkable differences in baseline data were found between the two groups (Table 1).

PPT was measured in 10 muscles on the body surface: levator scapulae and rhomboid muscles in the proximal part of the body; iliopsoas, quadratus lumborum, erector spinae, transverse abdominis, gluteus maximus, and piriformis in the waist; and hamstrings and gastrocnemius muscles in the lower extremities. No considerable differences in PPTs at the 10 measured points were observed between the two groups (Table 2).

### 3.2. Differences in State-Trait Anxiety and Pittsburgh Sleep Quality Index

Compared with the asymptomatic group, the CLBP group had significantly higher state anxiety scores (41.64 ± 9.88 vs. 36.69 ± 8.31; *t* = −2.496, *p*=0.015). However, no significant difference in trait anxiety scores (44.00 ± 7.83 vs. 42.67 ± 9.51; *t* = −0.695, *p*=0.489) was found between the two groups (Table 3).

Significant difference in total Pittsburgh Sleep Quality Index scores (6.41 ± 2.43 vs. 5.09 ± 2.18; *t* = −2.628, *p*=0.010) was found between the two groups. The CLBP group had a significantly worse sleep disorder (1.41 ± 1.16 vs. 0.93 ± 0.39; *t* = −2.587, *p*=0.011) than the asymptomatic group. People with CLBP slept less (0.44 ± 0.64 vs. 0.20 ± 0.46; *t* = −1.961, *p*=0.053) and were more likely to have daytime dysfunction (1.90 ± 0.82 vs. 1.60 ± 0.69; *t* = −1.808, *p*=0.074) than asymptomatic people, but the difference was not significant. Hypnotics were consumed by the CLBP group but not by the asymptomatic group (0.08 ± 0.48 vs. 0.00 ± 0.00; *t* = −1.075, *p*=0.285). No significant differences in time to fall asleep (1.05 ± 0.86 vs. 1.16 ± 0.80; *t* = 0.578, *p*=0.565), sleep efficiency (0.28 ± 0.61 vs. 0.13 ± 0.41; *t* = −1.340, *p*=0.184), and sleep quality (1.26 ± 0.75 vs. 1.07 ± 0.65; *t* = −1.238, *p*=0.219) were observed between the two groups (Table 4).

### 3.3. Correlation among Anxiety, Sleep Quality, and PPT

State anxiety showed a moderate negative correlation with levator scapulae threshold in the CLBP group and low negative correlations with rhomboid, iliopsoas, quadratus lumborum, erector spinae, and transverse abdominis muscles. Trait anxiety had a moderate negative correlation with levator scapulae threshold in the CLBP group and low negative correlations with rhomboids, iliopsoas, quadratus lumborum, and erector spinae muscles. In the total sample size (CLBP + asymptomatic), the results of the correlation were similar to those for the CLBP group. No remarkable correlation was observed between the Pittsburgh Sleep Quality Index score and PPT (Table 5).

## 4. Discussion

In this study, the CLBP group had considerably higher state anxiety than the asymptomatic population, but no difference was found in trait anxiety. This finding suggests the similarities in the long-term personality traits between the CLBP and asymptomatic groups. Nevertheless, the anxiety state was affected by CLBP. Whether the persistence of pain state for a long time can affect both populations or is induced by the characteristics of intermittent CLBP episodes remains unclear. State anxiety is an independent predictor of CLBP [34]. Pain-related anxiety affects patients' pain perception. Patients have the potential to catastrophize pain [35], increase the associated negative experience, and intensify their willingness to seek medical care [36]. A low to moderate negative correlation was found between state-trait anxiety scale and PPT, and no significant correlation was observed for lower extremity muscles. This finding indicates that individuals with stronger anxiety levels have lower PPT and a stronger willingness to report pain. Patients with CLBP are more intolerant to persistent painful stimuli and report a larger area of pain than asymptomatic people [26]. Differences in psychological status affect the patient's experience of pain and decision-making for LBP in clinical practice, and patients without psychological disorders will achieve better outcomes than those experiencing these illnesses [37, 38].

The CLBP group had considerably worse sleep quality, sleep disturbance, and a certain degree of daytime dysfunction than the asymptomatic population. Only the CLBP group reported using hypnotic drugs, and no significant difference was observed between the two. These findings are similar to previous studies [39–41], which indicated that sleep disturbance in patients with CLBP is related to pain intensity; that is, every increase in the visual analog scale is associated with a 10% increase in the likelihood of reporting sleep disturbance [18, 42]. A weak association was observed between CLBP intensity and sleep disturbance, suggesting that other factors, such as anxiety, also contribute to sleep disturbance in patients with CLBP. A good night's sleep provides only temporary relief [39], suggesting that comprehensive interventions for CLBP should include sleep quality, pain assessment, and coping. Sribastav et al. [19] pointed out that the patients with CLBP who experienced sleep disturbance had more severe anxiety, depression, and pain intensity and worse quality of life than those without sleep disturbance. Here, sleep quality is remarkably correlated with LBP severity but not with PPT. This result may be related to the small sample size. Hence, further research on this correlation is necessary.

No remarkable differences in PPT were observed. Moreira's cross-sectional study [43] of 36 subjects found that PPT is substantially lower in patients with CLBP than in healthy subjects. Farasyn and Meeusen [44] conducted a cross-sectional analysis of 87 patients with CLBP and 64 healthy subjects and also found that PPT is considerably lower in patients with CLBP than in healthy subjects. O'Neill et al.study [45] analyzed 198 patients with CLBP and 44 controls and found that PPT and other pain sensitivity parameters are considerably lower in patients with CLBP than in the controls. Other studies [29, 46] reported similar results. The discrepancy in findings may be attributed to the differences between the definition for the control and asymptomatic populations and other factors, such as sample size and the subject's age. The present results can only reflect the objective data of the samples in this study.

This study has limitations. It has a cross-sectional correlation design, and the subjects were relatively young (mostly 22 years). Subsequent studies should expand the sample size and add subjects from other age groups to produce representative results.

## 5. Conclusion

Patients with CLBP have considerably worse state anxiety and sleep quality than asymptomatic people; however, no remarkable difference in PPT was found between the two groups. State anxiety and trait anxiety showed a low to moderate negative correlation with PPT, and no remarkable correlation was observed between the Pittsburgh Sleep Quality Index score and PPT. The results suggest that in clinical practice, the focus should include pain and related social and psychological factors. CLBP treatment should be considered from multiple perspectives and disciplines.

## Figures and Tables

**Table 1:** Baseline data (*M* ± SD).

	Chronic low back pain (*n* = 40)	Asymptomatic (*n* = 40)	*t*	*p*
Age (years)	22.45 ± 2.36	21.55 ± 2.09	−1.805	0.075
Male (*n* (%))	20 (25.00)	20 (25.00)	None	None
Female (*n* (%))	20 (25.00)	20 (25.00)		
Height (cm0	170.48 ± 9.56	169.05 ± 9.82	−0.658	0.513
Weight (kg)	62.03 ± 12.87	61.51 ± 11.45	−0.188	0.851
Education (years^&^)	4.43 ± 1.82	4.08 ± 1.93	−0.834	0.407
Sedentary (*n* (%))	22 (27.50)	26 (32.50)	−0.906	0.368
Regular training (*n* (%))	27 (33.75)	22 (27.50)	1.142	0.257
Duration of each training (mins)	61.88 ± 37.84	55.00 ± 38.81	−0.802	0.425
Frequency (times per week)	3.33 ± 1.75	2.73 ± 1.81	−1.509	0.135
Self-perceived intensity^+^	2.78 ± 1.17	2.65 ± 1.25	−0.462	0.645

^&^Freshman was 1, sophomore was 2, junior year was 3, senior year was 4, the first year of postgraduate was 5, and so on. +: 0–6 points; 0, rest; 1, very weak; 2, mild; 3, medium; 4, tiredness; 5 very tired.

**Table 2:** Differences in the PPT between patients with CLBP and asymptomatic people (*M* ± SD; units: kgf).

	Chronic low back pain (*n* = 40)	Asymptomatic (*n* = 40)	*t*	*p*
Levator scapula	4.08 ± 1.61	4.07 ± 1.43	−0.035	0.972
Rhomboid muscle	4.79 ± 1.78	4.45 ± 1.43	−0.981	0.330
Iliopsoas	5.42 ± 2.06	5.07 ± 1.79	−0.825	0.412
Quadratus lumborum	6.24 ± 2.16	6.03 ± 2.24	−0.449	0.654
Erector spinae	7.80 ± 2.50	7.05 ± 2.33	−1.422	0.159
Transversus abdominis	5.82 ± 2.14	5.37 ± 2.07	−0.973	0.333
Gluteus medius	6.28 ± 1.79	5.90 ± 1.86	−0.954	0.343
Piriformis	6.63 ± 2.07	6.45 ± 2.19	−0.391	0.697
Hamstring muscle	6.74 ± 2.46	6.48 ± 2.15	−0.532	0.596
Gastrocnemius	5.33 ± 1.44	5.39 ± 1.55	0.182	0.856

**Table 3:** Differences in the state-trait anxiety inventory scores between patients with CLBP and asymptomatic people (*M* ± SD).

	Chronic low back pain (*n* = 40)	Asymptomatic (*n* = 40)	*t*	*p*
State anxiety	41.64 ± 9.88	36.69 ± 8.31	−2.496	**0.015** ^*∗*^
Trait anxiety	44.00 ± 7.83	42.67 ± 9.51	−0.695	0.489

^*∗*^ *p* < 0.05.

**Table 4:** Differences in the Pittsburgh Sleep Quality Index between patients with CLBP and asymptomatic people (*M* ± SD).

	Chronic low back pain (*n* = 40)	Asymptomatic (*n* = 40)	*t*	*p*
Time to fall asleep	1.05 ± 0.86	1.16 ± 0.80	0.578	0.565
Sleeping time	0.44 ± 0.64	0.20 ± 0.46	−1.961	0.053
Sleep efficiency	0.28 ± 0.61	0.13 ± 0.41	−1.340	0.184
Sleep disorder	1.41 ± 1.16	0.93 ± 0.39	−2.587	**0.011** ^*∗*^
Sleep quality	1.26 ± 0.75	1.07 ± 0.65	−1.238	0.219
Hypnotics	0.08 ± 0.48	0.00 ± 0.00	−1.075	0.285
Daytime dysfunction	1.90 ± 0.82	1.60 ± 0.69	−1.808	0.074
Total score	6.41 ± 2.43	5.09 ± 2.18	−2.628	**0.010** ^*∗*^

^*∗*^ *p* < 0.05.

**Table 5:** Correlation among anxiety, sleep quality, and PPT in patients with chronic low back pain.

			LS	RHO	ILI	QL	ES	TRA	GM	PIR	HM	GAS
Chronic low back pain (*n* = 40)	State anxiety	Pearson	−0.433	−0.335	−0.386	−0.360	−0.403	−0.379	−0.298	−0.182	−0.303	−0.291
		*p* value	**0.006** ^*∗*^	**0.037** ^*∗*^	**0.015** ^*∗*^	**0.024** ^*∗*^	**0.011** ^*∗*^	**0.017** ^*∗*^	0.065	0.268	0.060	0.072
	Trait anxiety	Pearson	−0.465	−0.335	−0.333	−0.338	−0.390	−0.272	−0.284	−0.120	−0.278	−0.279
		*p* value	**0.003** ^*∗*^	**0.037** ^*∗*^	**0.038** ^*∗*^	**0.035** ^*∗*^	**0.014** ^*∗*^	0.094	0.080	0.467	0.087	0.086
	Pittsburgh sleep quality index	Pearson	−0.014	0.103	0.071	0.142	0.132	0.079	0.111	0.204	0.179	0.089
		*p* value	0.933	0.533	0.667	0.390	0.423	0.631	0.502	0.212	0.277	0.589
Chronic low back pain + asymptomatic (*n* = 80)	State anxiety	Pearson	−0.346	−0.222	−0.248	−0.250	−0.263	−0.234	−0.175	−0.136	−0.196	−0.166
		*p* value	**0.001** ^*∗*^	**0.043** ^*∗*^	**0.023** ^*∗*^	**0.022** ^*∗*^	**0.016** ^*∗*^	**0.032** ^*∗*^	0.111	0.216	0.074	0.132
	Trait anxiety	Pearson	−0.353	−0.234	−0.178	−0.244	−0.267	−0.193	−0.142	−0.072	−0.185	−0.161
		*p* value	**0.001** ^*∗*^	**0.032** ^*∗*^	0.106	**0.026** ^*∗*^	**0.014** ^*∗*^	0.078	0.199	0.518	0.093	0.143
	Pittsburgh sleep quality index	Pearson	−0.098	0.030	−0.025	−0.031	−0.009	−0.019	−0.006	0.049	0.074	0.003
		*p* value	0.375	0.786	0.819	0.781	0.935	0.861	0.957	0.658	0.506	0.980

LS: levator scapula; RHO: rhomboid; ILI: iliopsoas; QL: quadratus lumborum; ES: erector spinae; TRA: transversus abdominis; GM: gluteus medius; PIR: piriformis; HM: hamstring muscle; GAS: gastrocnemius. ^*∗*^*p* < 0.05.

## Data Availability

The datasets are available from the corresponding author on reasonable request.
